# Changes in conception rates, not in pregnancy‐related behaviour, likely caused decline in preterm births during the first year of the COVID‐19 pandemic

**DOI:** 10.1111/1471-0528.17568

**Published:** 2023-06-04

**Authors:** Peter Fallesen, Moritz Oberndorfer, Marco Cozzani

**Affiliations:** ^1^ Swedish Institute for Social Research Stockholm University Stockholm Sweden; ^2^ Rockwool Foundation Copenhagen Denmark; ^3^ Institute of Social Medicine, Centre for Public Health Medical University of Vienna Vienna Austria; ^4^ MRC/CSO Social and Public Health Sciences Unit University of Glasgow Glasgow UK; ^5^ Population Research Unit University of Helsinki Helsinki Finland; ^6^ Population and Society Research Unit, Department of Statistics, Computer Science, Application ‘Giuseppe Parenti’(DiSIA) University of Florence Florence Italy

A series of studies, including recent work published in BJOG by Rusconi et al.,^1^ has highlighted the surprising fact that measures of birth outcomes such as preterm birth and low birthweight improved 9 months after the first wave (February–June 2020) of the COVID‐19 pandemic across countries such as Italy, Spain, Ireland and the USA (see Yang et al.^2^ for recent meta study). This growing body of work has generated important knowledge about the children conceived and born during the pandemic, and how they as a cohort may differ from those born before and after the pandemic. Yet, we believe that this strand of literature has overlooked key demographic drivers behind the changes in birth outcomes observed during the pandemic. Here we describe why an improvement in birth outcomes observed during the pandemic is likely caused by decline in conception rate and changing selection into conception in the months following the onset of the pandemic rather than changing behaviours among pregnant women during the pandemic.

In their *BJOG* article, Rusconi et al.^1^ (p. 282) find that rates of preterm births declined drastically in September–November 2020 relative to trend, and they ask researchers to consider what lessons the pandemic may teach about the ‘possible importance of lifestyle and environmental aspects related to the occurrence of pregnancies ending preterm’. Others have noted the non‐causal nature of these studies,^3^ which should be kept in mind for future research to cover. We, however, suggest a more fundamental aspect of the pandemic's effect on pregnancies has been overlooked broadly in the literature, which can account for most, if not all, of the decline found by Rusconi et al. and many other studies. The cause of decline in preterm births and other adverse birth outcomes should not necessarily be sought in changing behaviour or services during pregnancy, but rather in changes in how many and who conceived during the early stages of the pandemic,^4^, ^5^ as declines in conceptions and subsequent fertility rates have been observed across most developed countries.^6^

Figure 1 shows the monthly crude birth rate (CBR; number of births per 1000 population) for Italy for 2020 and 2021 measured relative to the 2019 monthly CBR. As clearly seen from the figure, the number of births declined drastically relative to 2019 starting September 2020 and until January 2021, with the CBR in January 2021 being 14% lower than what was observed in January 2019, which is the equivalent of 8.4 fewer births per 100 000 population that month. Rapidly declining birth rates are mostly caused by rapidly declining conception rates and are unlikely to be only explained by potential changes in pregnancy loss, abortion, maternal emigration or stillbirth rates. Mechanically, a decline in conceptions will manifest first as a decline in preterm births 7–8 months after the number of conceptions dropped.^4^ This is because (fewer) preterm babies, which were conceived after the pandemic onset, are born at the same time as full‐term babies conceived before the COVID‐19 pandemic. Thus, the decline in preterm rates can be seen as a demographic artefact caused by declining conceptions without any change to pregnancy‐related behaviour occurring. In recent work, we have shown that a similar trend in fertility can be observed in Spain for the same period.^4^, ^7^ In this work, we also show a similar decline in Spanish preterm births rates as observed in Italy by Rusconi et al.^4^ Further, we demonstrate how this decline in preterm births can occur mechanically in the case of a rapid decline in conceptions right after the onset of the COVID‐19 pandemic, which in turn leads to the observed lower birth rates beginning by September (fewer preterm births) and carrying out all through to January 2021 (fewer term births).

**FIGURE 1:**
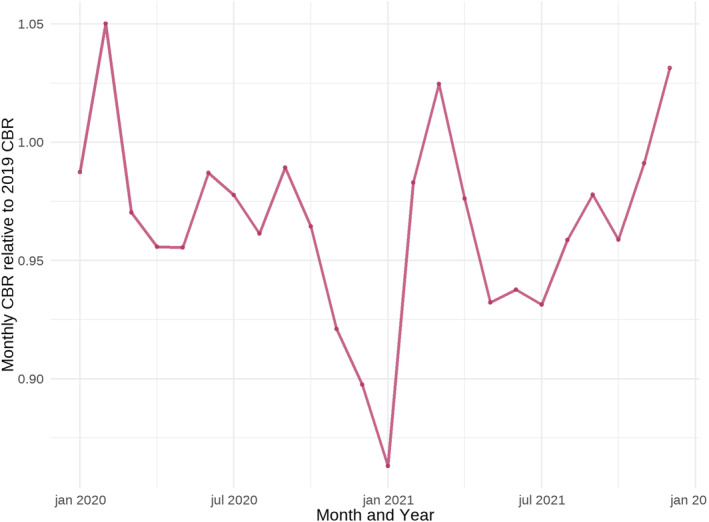
Monthly crude birth rate for Italy 2020–2021 measured relative to the monthly crude birth rate in 2019. *Source*: ISTAT.

Further, we also analyse changes in who conceives. In the Spanish case the data allow us to examine which groups see the largest conception declines. In relative terms, the two groups that see the largest decline are women at the beginning and end of the reproductive age – the two groups also at highest risk of giving birth to preterm babies because of, respectively, precarious and unplanned pregnancies occurring among the young^8^ and higher rates of complications and medically assisted reproductive (MAR) conceptions among older women.^9^ During the first COVID‐lockdown, young people's risk for precarious and unplanned pregnancies declined drastically due to stay‐at‐home orders, and MAR clinics shut down services. Moreover, the COVID‐19 pandemic has likely led to changes in the composition of parents in regard to other characteristics known to be associated with preterm birth.^5^ For example, initial evidence is emerging that babies conceived in the Global North during the pandemic have, on average, more socio‐economically advantaged parents compared with babies conceived before the COVID‐19 pandemic.^5^, ^10^ More advantaged parental socio‐economic circumstances, in turn, have consistently been shown to be associated with a lower probability of preterm birth.^11^ Pandemic‐induced compositional shifts in parental characteristics provide us with another plausible explanation for improved birth outcomes during the COVID‐19 pandemic in the Global North, whereas the situation in countries with less universal access to contraceptive measures may have seen different developments, as suggested by Pesando & Abufhele^12^ for Chile.

To conclude, the COVID‐19 pandemic may have generated a pure demographic artefact driven by a population‐wide decline in conceptions discussed above as well as heterogeneous conceptive responses across the affected populations. These two differential effects of the COVID‐19 pandemic may also explain why babies conceived during the pandemic show improved birth outcomes compared with babies conceived before the pandemic. When interpreting the COVID‐19 consequences on newborn health we thus advise disentangling the direct effect of in utero exposure to the COVID‐19 pandemic and the consequences of lockdown measures on birth outcomes from a demographic artefact and pandemic‐induced changes in the composition of who became pregnant. Importantly, the latter determinants and the former ones may have different clinical implications for the population of newborns and their long‐term development trajectories.

## AUTHOR CONTRIBUTIONS

PF, MO and MC conceived, planned, carried out, analysed and wrote up the paper. All authors approved final version.

## FUNDING INFORMATION

PF's work was supported by ROCKWOOL Foundation (1227) and the Swedish Research Council for Health, Working Life and Welfare (2016‐07099). MO's work was supported by the Medical Research Council (MC_UU_00022/2) and the Scottish Government Chief Scientist Office (SPHSU17). MC's work was supported by the Italian Ministry of the University and Research (PE8‐B83C22004800006).

## CONFLICT OF INTEREST STATEMENT

None declared.

## ETHICS APPROVAL

Only publicly available secondary data were used for analysis and therefore are exempt from ethical approval.

## Data Availability

The data that support the findings of this study are available in ISTAT at http://dati.istat.it/. These data were derived from the following resources available in the public domain: ISTAT, http://dati.istat.it/.
